# Fast genetic mapping using insertion-deletion polymorphisms in *Caenorhabditis elegans*

**DOI:** 10.1038/s41598-021-90190-x

**Published:** 2021-05-26

**Authors:** Ho-Yon Hwang, Jiou Wang

**Affiliations:** Department of Biochemistry and Molecular Biology, Department of Neuroscience, Johns Hopkins University, 615 N. Wolfe Street, E8410, Baltimore, MD 21205 USA

**Keywords:** Biological techniques, Computational biology and bioinformatics, Developmental biology, Genetics, Molecular biology, Neuroscience

## Abstract

Genetic mapping is used in forward genetics to narrow the list of candidate mutations and genes corresponding to the mutant phenotype of interest. Even with modern advances in biology such as efficient identification of candidate mutations by whole-genome sequencing, mapping remains critical in pinpointing the responsible mutation. Here we describe a simple, fast, and affordable mapping toolkit that is particularly suitable for mapping in *Caenorhabditis elegans*. This mapping method uses insertion-deletion polymorphisms or indels that could be easily detected instead of single nucleotide polymorphisms in commonly used Hawaiian CB4856 mapping strain. The materials and methods were optimized so that mapping could be performed using tiny amount of genetic material without growing many large populations of mutants for DNA purification. We performed mapping of previously known and unknown mutations to show strengths and weaknesses of this method and to present examples of completed mapping. For situations where Hawaiian CB4856 is unsuitable, we provide an annotated list of indels as a basis for fast and easy mapping using other wild isolates. Finally, we provide rationale for using this mapping method over other alternatives as a part of a comprehensive strategy also involving whole-genome sequencing and other methods.

## Introduction

Forward genetics using randomly generated mutations and mutants has traditionally driven genetic analysis in *Caenorhabditis elegans*^1^. Establishing linkage between a mutation and the corresponding mutant phenotype is important in forward genetics. To help establish linkage, mapping could be done to place a mutation within a small interval, and rescue experiments^2^ could further narrow the interval. Sanger DNA sequencing^3^ has long been used to identify candidate mutations while whole-genome sequencing^4,5^ has simplified the identification of candidate mutations. A candidate mutation needs to be confirmed, which could involve reiteration of the same mutant phenotype by mutations in the same gene using mutagenesis perhaps CRISPR/Cas9 or possibly by RNA-mediated interference (RNAi), complementation test, and rescue experiments. Even with increasing popularity of reverse genetic engineering methods, such as CRISPR/Cas9 in its early^6–14^ and later iterations^15–19^ and RNAi^20–24^, forward genetics is a key to new discoveries and remains important.

Preferred methods of mapping have changed with emerging technologies. With advances in large-scale DNA sequencing, use of DNA sequence polymorphisms have supplemented and largely replaced many traditional methods^25^, including deficiency mapping and classic 2-point and 3-point mapping using mutations that cause a visible mutant phenotype. Many popular methods use wild isolate CB4856^26^ from Hawaii (HA) with a large number of single nucleotide polymorphisms (SNPs) between CB4856 HA and the standard wild-type strain N2 Bristol. Initially, a special class of SNPs that lead to the creation or destruction of restriction sites were exploited^27^, and this method was simplified further by only using 48 SNPs that affect DraI restriction sites throughout the genome^28^. SNP-comparative genome hybridization (SNP-CGH) method^29^ uses ~ 76,000 SNP probes and allows a very high-resolution mapping, but this method is expensive and requires specialized material, equipment, and highly purified genomic DNA. Alternatively, whole-genome sequencing could be combined with mapping^30^, which could involve the use of CB4856 HA strain^31^ or could involve outcrossing mutations introduced by mutagenesis and dispense with CB4856^32^.

Here, we describe a distinct group of insertion-deletion polymorphisms (indels), specifically indels with length change from 40 to 699 base pairs (bp). While indels have been used for mapping in the past with *C. elegans*^33,34^ and with *C. briggsae*^35^, indels of 40 to 699 bp (i40-699) provide notable advantages. There are many i40-699 indels, which allows fairly high-resolution mapping, in 40 wild isolates whole-genome sequenced by the *C. elegans* million mutation project (MMP)^36^. Next, i40-699 indels could be identified quickly and easily by polymerase chain reaction (PCR) simply followed by agarose gel electrophoresis. Furthermore, we optimized the primers for detecting i40-699 indels so that PCR could be performed without DNA purification. This optimization improves the speed and ease of mapping because there is no need to spend several days growing many large populations of worms. No need for many worms also allows easier mapping with mutations causing severe biological consequences such as sterility. We describe examples of mapping using previously known and unknown mutations. While we focus on i40-699 indels between N2 wild type and CB4856 HA, i40-699 indel mapping could be done instead using similar indels present in other *C. elegans* wild isolates sequenced by the MMP. Finally, we present ideas for the use of i40-699 indel mapping in conjunction with current techniques in forward genetics.

## Methods and materials

*C. elegans* strains were cultured using standard methods^1^. Strains used to initiate this study are: CB1033 *che-2(e1033)*, CB3329 *che-10(e1809)*, IW523 *che-10(iw109); daf-3(iw108)*, CB3241 *clr-1(e1754)*, CB1376 *daf-3(e1376)*, VC20208 without *daf-3(gk269916)*, VC20379 *daf-3(iw108)*, RB2589 *daf-3(ok3610)*, SD378 *dpy-17(e164) unc-79(e1068) / mpk-1(ga117)*, JN554 *dyf-11(pe554)*, PR813 *osm-5(p813)*, VC40961 *rund-1(gk901813)*, MT7554 *sqv-3(n2842) unc-69(e587)/qC1*, MT9647 *unc-29(e1072) sqv-5(n3039) / hT2*, PR691 *tax-2(p691); che-2(iw107)*, RB1546 *tmc-1(ok1859)*, VC40425 *tmc-1(gk631913)*, CB4856 Hawaiian HA wild isolate, and N2 wild-type strain. Additional strains were made using these strains.

The i40-699 indels from WormBase^37^ version WS256 natural variant data were extracted using custom R^38^ scripts in a separate study^39^. We also examined the WormBase version WS276 data to ensure that WS256 data is up-to-date. Using additional custom R scripts, we identified the nearest gene corresponding to each i40-699 indel along with the genetic and physical position of the gene using WormBase annotated gene dataset. A list of 11,556 annotated i40-699 indels are in Supplementary Table S1 with the physical position and the size of the indels, nearest gene with their physical and genetic position, frequency of appearance among the 40 wild isolates, and wild isolates with the i40-699 indel.

In designing primers, we aimed for uniform PCR condition and easy distinction of N2 and CB4856 HA DNA. For example, we chose i40-699 indels with longer N2 DNA than CB4856 for easy distinction. For identical annealing temperature, optimum melting temperature Tm was set at 60 °C. For identical elongation time, we kept the sizes of N2 and CB4856 products within a narrow range. With the final primer set, N2 product sizes are between 500 and 1500 bp whereas CB4856 product sizes are between 400 and 1300 bp. For easier separation of PCR products, we usually picked indels of > 100 bp length change, but we picked few indels of < 100 bp length change to have a representative i40-699 indel in most megabase (Mb) intervals. Otherwise, we picked i40-699 indels arbitrarily. To minimize confusing PCR products amplified from another part of the genome, primer-BLAST^40^ was performed initially using the six *C. elegans* chromosome sequences as the template with specificity check against the *C. elegans* non-redundant (nr) nucleotide database. Additional primers were selected using primer-BLAST again or using primer3^41^ with the appropriate cosmid, fosmid or YAC clone sequence, and we tested the primers until a satisfactory primer pair was identified. A total of 584 primers were tested, and the best primer pairs are listed in Supplementary Table S2 with expected sizes of the PCR products, PCR success rates under two different conditions, annotations of the position in 96-well plate where applicable, and wild isolates with the i40-699 indel.

Working stocks of premixed primer pairs were stored in 96-well plates frozen because long-term storage at 4 °C led to evaporation and possible degradation. Unless indicated otherwise, adult hermaphrodites were lysed individually in 10 μl of 1 × lysis buffer (40 mM KCl, 10 mM Tris pH 8.3, 2.5 mM MgCl_2_, 0.45% IGEPAL, 0.45% Tween 20) with proteinase K added prior to lysis at 60 μg/ml final concentration. Lysis was performed at 65 °C for 1 h with inactivation at 95 °C for 15 min using 8-strip tubes. Lysed worms were often mixed or pooled, and combined samples were often diluted with water or 1 × lysis buffer lacking proteinase K. PCR was performed in 25 µl volume using standard conditions without detergent for Taq polymerase. Either 8-strip tubes or 96-well plates were used for PCR with aluminum foil seal for 96-well plates to reduce evaporation. The PCR temperature cycler condition was: 5′ 95 °C, 35 cycles of (30″ 95 °C, 30″ 60 °C, 2′ 72 °C), 5′ 72 °C. PCR products were visualized after separation by electrophoresis for 40 min at 120 V on 2% agarose gel with ~ 1.5 l of Tris–acetate-EDTA running buffer unless indicated otherwise. Recurring poor PCR results were usually rectified by new preparation of 1 × lysis buffer and discarding old stocks of 1 × lysis buffer, which could become less effective after many months.

To obtain F2 mutants for mapping, CB4856 males were mated with mutant hermaphrodites of interest. Next, F1 heterozygous hermaphrodites were placed in new plates as L4 larvae to reproduce F2 progeny by self-fertilization. Finally, F2 homozygous mutants were identified by their mutant phenotype, which could be clear (Clr) and blistering body^42^, squashed vulva (Sqv) at L4 larval stage^43^ and associated sterility with a stereotypical morphology of embryos incapable of cytokinesis^44^, or a temperature-dependent constitutive dauer mutant phenotype^45^. Collection of temperature-dependent dauer mutants involved placing a mixed population of embryos and L1 larvae at 28 °C for 10 days in the presence of plenty of *E. coli* bacteria serving as food prior to a return to 20 °C to allow mutant dauer survivors to become mutant fertile adults.

Complementation tests using the temperature-dependent constitutive dauer mutant phenotype were performed using a few different approaches. For *iw108* located in chromosome X, either *daf-3(ok3610)* or *dyf-11(pe554)* mutant hermaphrodites were mated with N2 males, and F1 hemizygous mutant XO males were mated with either *daf-3(iw108)* or *dpy-17(e164) unc-79(e1068); daf-3(iw108)* mutant hermaphrodites. Mixed populations of F2 progeny were moved to 28 °C for 10 days and subsequently were moved to 20 °C, and non-Dpy non-Unc fertile adult hermaphrodites were sought in the presence of alive males. Dauer survivors that became adult males were used to assess successful mating with the absence of fertile hermaphrodites indicating no complementation. With tests using *daf-3(iw108)* hermaphrodite mutants without *dpy-17* or *unc-79* mutations, dauer survivors that became fertile adult hermaphrodites were examined using PCR to confirm the presence or absence of the mutations of interest. Similar approach was used with *iw107* also located in chromosome X. Here, one of *che-2(e1033)*, *daf-3(ok3610)*, *dyf-11(pe554)*, *rund-1(gk901813)*, *osm-5(p813)*, or *iw107* mutant hermaphrodites were mated with N2 males, and F1 hemizygous mutant males were mated with one of *che-2(iw107)*, *dpy-17(e164) unc-79(e1068); che-2(iw107)*, *dpy-17(e164) unc-79(e1068); che-2(e1033)*, *dpy-17(e164) unc-79(e1068); dyf-11(pe554)*, or *dpy-17(e164) unc-79(e1068); osm-5(p813)* hermaphrodites. A different approach was used with *iw109* located in chromosome II. Here, *che-10(e1809)* mutant hermaphrodites were mated with N2 males, and heterozygous F1 males were mated with either *che-10(iw109)* or *dpy-17(e164) unc-79(e1068); che-10(iw109)* mutant hermaphrodites. After 10 days at 28 °C, the presence of dauer survivors that became males was used as evidence of complementation.

Whole genome sequencing of *che-10(iw109)* mutant strain was performed with Novogene (en.novogene.com), using their Plant and Animal Whole Genome Sequencing service with 2 Gb data output for 20 × coverage of the genome by paired-end 150 bp sequencing. Sanger sequencing was performed with Genewiz (www.genewiz.com).

## Results

### Easily distinguished indels in CB4856

**Figure 1:**
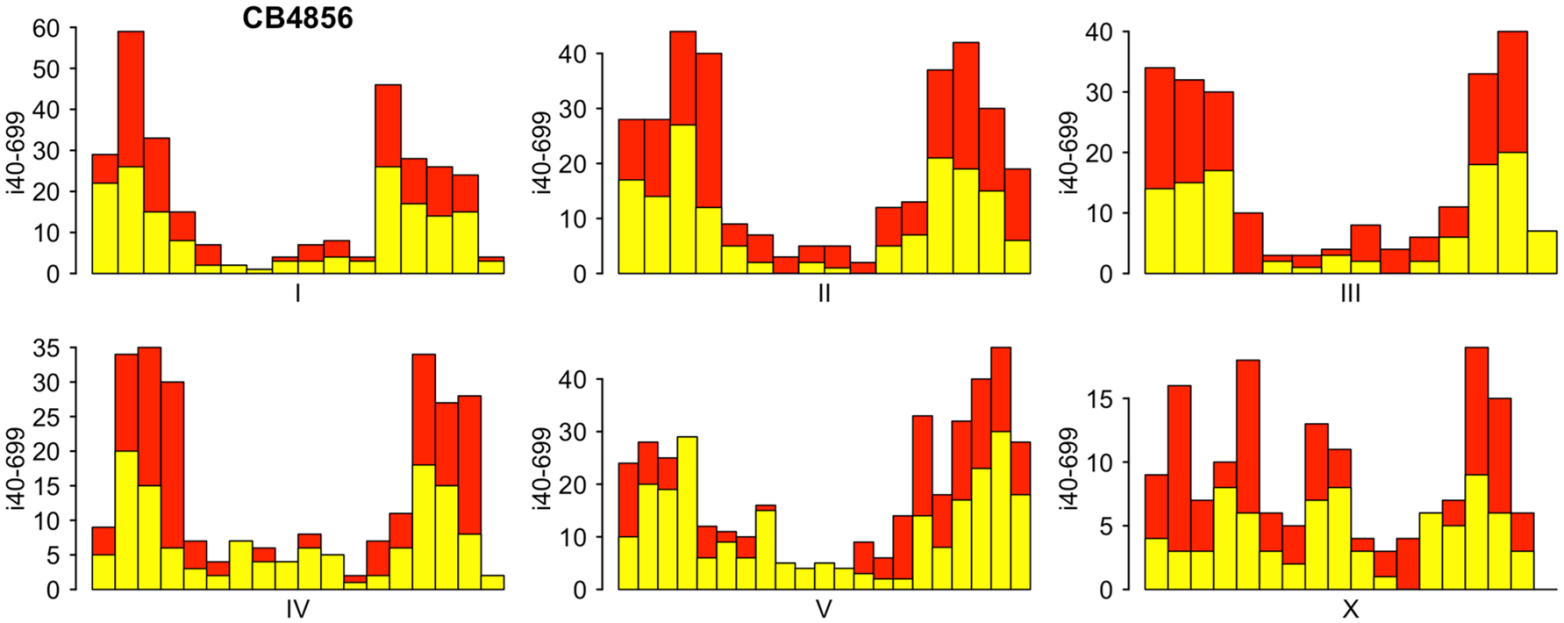
Distribution of i40-699 indels in CB4856. Shown is distribution of i40-699 indels in 1 Mb intervals across six chromosomes of CB4856. Red bars indicate i40-699 indels that are also present in at least one of the other 39 wild isolates. Yellow bars indicate i40-699 indels that are unique to CB4856 among the 40 wild isolates.

Indels of a certain range of DNA length change, for example from 40 to 699 bp, could serve as excellent mapping resource. Mapping with i40-699 indels could be done with PCR followed by gel electrophoresis without restriction enzyme digest. From over 230,000 indels identified by the MMP in 40 wild isolates^36^, we picked and annotated over 11,000 i40-699 indels (Supplementary Table S1). CB4856 HA wild isolate, which is commonly used by many laboratories for mapping, contains over 1,600 i40-699 indels. These i40-699 indels are distributed throughout the genome in an uneven manner with central regions of autosomal chromosomes having far fewer i40-699 indels than autosomal chromosome arms (Fig. 1), as is typical in wild populations for all SNPs and indels^39^. Unique among the 40 wild isolates, CB4856 has at least one i40-699 indel within every interval of one Mb. Given these advantages, we picked i40-699 indels in CB4856 for mapping.

To confirm the presence of i40-699 indels, we examined 124 i40-699 indels by PCR with 586 primers (Supplementary Table S3). All PCR was performed using lysed worms without DNA purification. We obtained PCR products of expected sizes for all but three loci with both CB4856 and N2, thus confirming the existence of most i40-699 indels. With two unconfirmed i40-699 indels WBVar02075736 and WBVar02075902, PCR products corresponding to N2 were obtained but not CB4856. We do not consider the absence of CB4856 products as a proof that i40-699 indel is absent because PCR failure could be caused by altered primer-binding site, which could be a consequence of reported or unreported mutation. We subsequently examined six primer binding sites used for successful PCR of these two indel loci and identified two SNPs affecting 5′ end and middle of two binding sites for WBVar02075902 in CB4856, but PCR is often possible with such minor DNA changes. As for the third problematic locus with two closely neighboring i40-699 indels, the larger of the two indels WBVar02076224 appeared to be absent while the status of the smaller indel WBVar02091533 was inconclusive. Thus, false positive rate for i40-699 indel calling by the MMP seems low (< 1%) with CB4856, but reported and unreported differences in the DNA sequences flanking the i40-699 indel could pose problems with primer design for indel detection.

**Figure 2:**
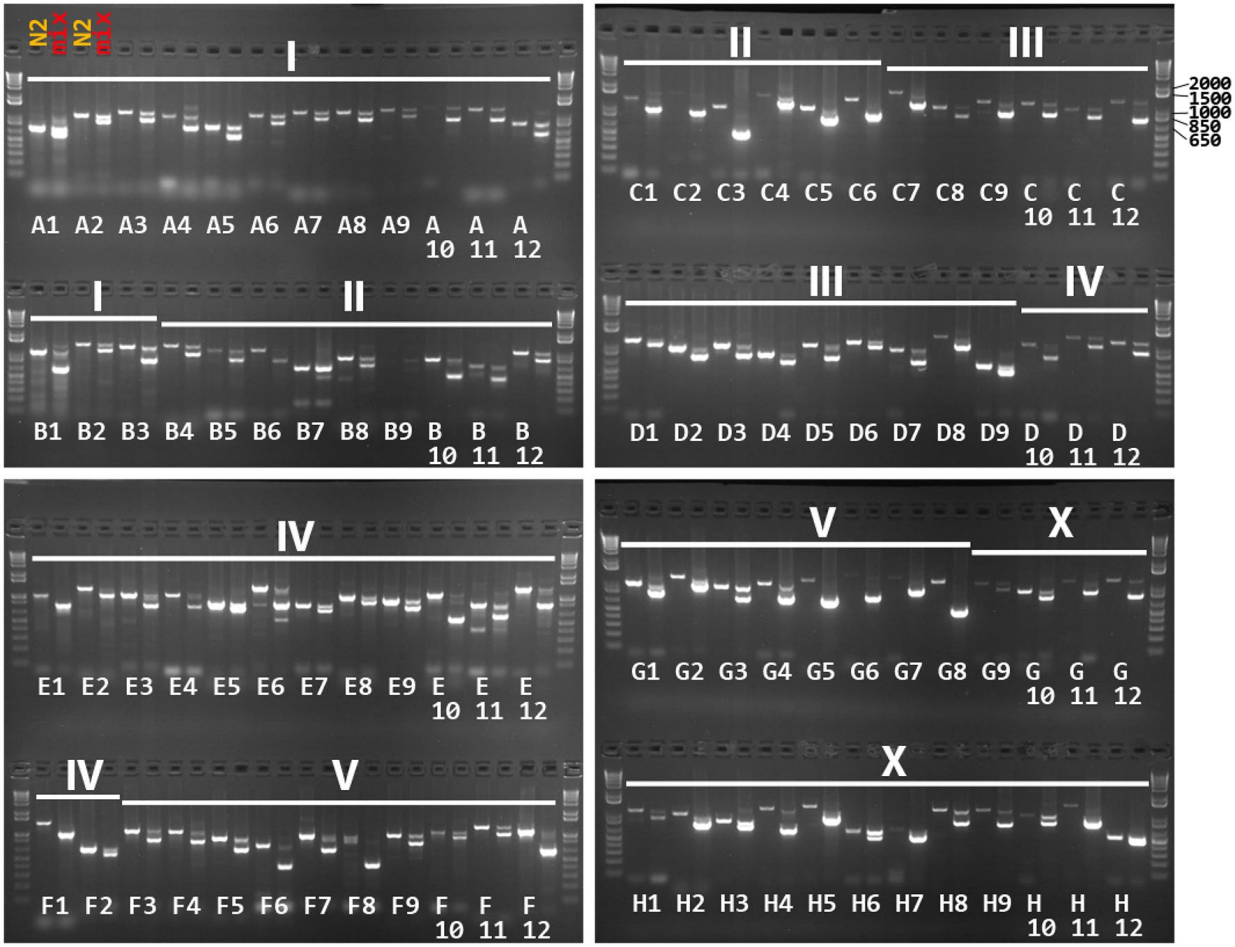
PCR using the primer set for detecting 96 i40-699 indels. Pairs of PCR products using DNA from N2 wild type on left and from equal parts N2 and CB4856 on right. Each PCR sample used 0.1 adults as template. The samples are arranged in 8 rows from A to H with each row containing 12 different primer pairs. Shorthand designations from A1 to A12 through from H1 to H12 indicate primer pair positions in a 96-well plate. Chromosomes are indicated as I, II, III, IV, V, and X on top. 1 kb Plus Ladder Invitrogen.

Next, we selected primers capable of detecting i40-699 indels reliably with a fraction of a worm. Here, 0.1 adult hermaphrodites were used for each PCR sample to test the primers using 96-well PCR plates, and unsatisfactory primers with a variety of complications were abandoned. Targeting 102 different i40-699 indel loci, PCR products of expected sizes were obtained at > 90% rate with 95 primer pairs (Supplementary Table S2). To serve as a convenient reference, a set of 2% agarose gel images of PCR products using either N2 or an equal mix of N2 and CB4856 with 96 primer pairs is shown in Fig. 2. In another test, PCR was performed using a mix of 0.01 CB4856 adults and 0.09 N2 adults per sample. Here, PCR products corresponding to CB4856 were obtained at ≥ 75% rate with 82 primer pairs, and 100% success rate associated with nearly half of the primer pairs (Supplementary Table S2). A set of 2% agarose gel images of PCR products using 1-to-9 mix of CB4856 and N2 is provided in Supplementary Figure S1. Thus, our primers could be reliably used without needing several days to grow large populations of worms and without DNA purification.

### Validation mapping with known and unknown mutations

**Figure 3:**
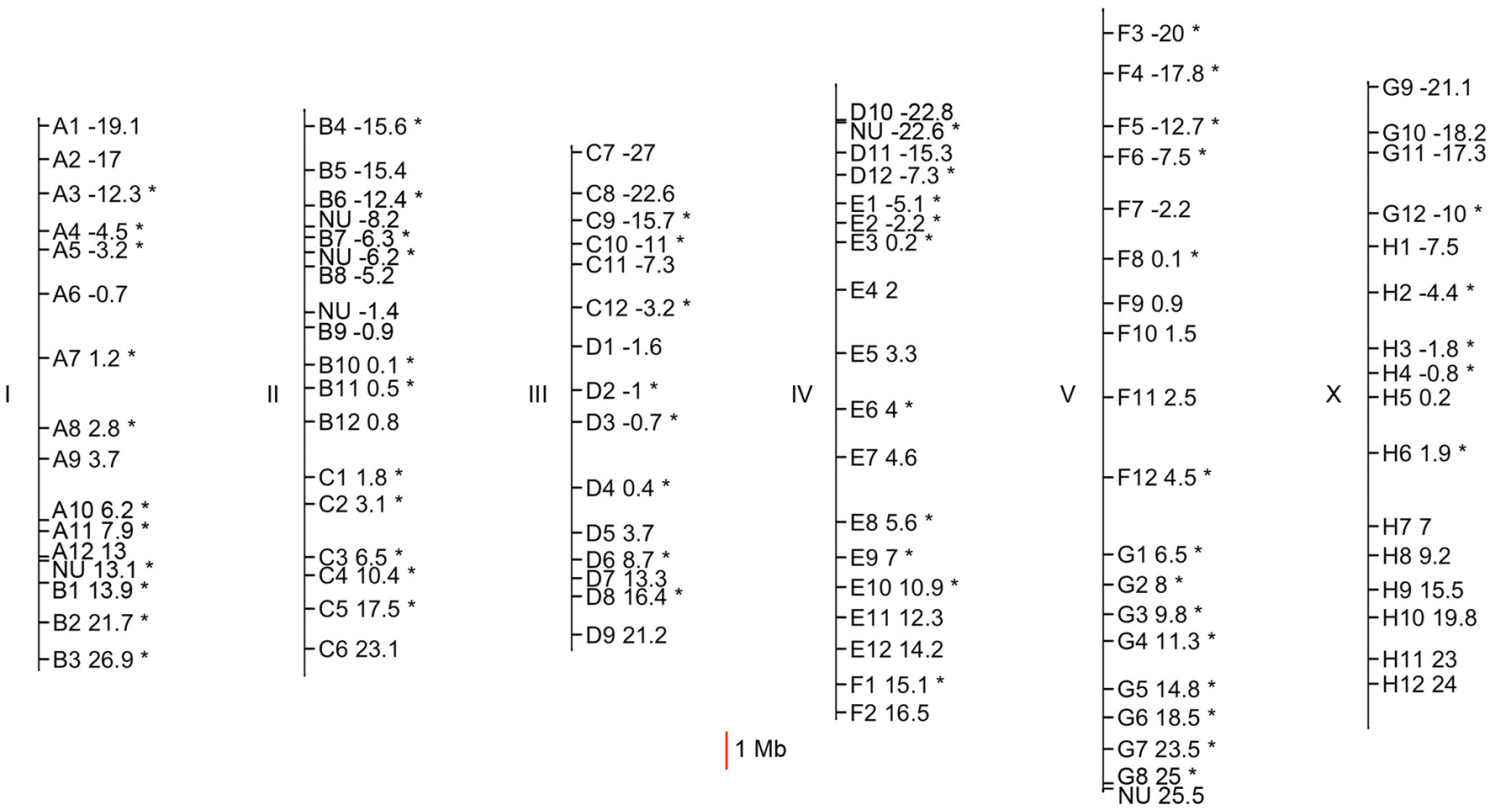
Location of 102 i40-699 indels with good primers. Six *C. elegans* chromosomes are depicted with their left ends on top. Each i40-699 indel is marked by their position in the 96-well plate (e.g. A1 to H12) used for most mapping experiments or is marked as NU if not a part of the oft-used 96-well plate. Physical location of indel is marked with a notch, and genetic position is indicated with a number. Indels on the left side of each chromosome have negative number for genetic position. Indels with primer pairs showing 100% PCR success rate with 1:9 dilution of CB4856 and N2 are marked with star(*).

Common 96-well plate format was used for ease in quickly performing all experimental steps under identical conditions. We arranged the placement of primers in a 96-well plate from chromosome I to chromosome X and from left to right end of each chromosome (Fig. 3). Equipment needed is same as or less stringent than the older SNP mapping methods^27,28^ prior to the SNP-CGH method^29^, including multi-channel pipettor, temperature cycler capable of using 96-well plates, and agarose gel electrophoresis apparatus. As is standard, genetic manipulations start with CB4856 Hawaiian males mating with mutant hermaphrodites to produce heterozygous F1 animals. Homozygous F2 mutants from F1 heterozygotes are identified based on their mutant phenotypes. To make analysis easy, we performed PCR and gel electrophoresis using lysed N2 wild type worms in parallel with pooled F2 mutants. With an i40-699 indel closely linked to the mutation of interest, we expect only one PCR product corresponding to N2 in most trials. On the other hand with an unlinked i40-699 indel, we expect two PCR products corresponding to CB4856 and to N2. Because we designed CB4856 products to be smaller than the corresponding N2 products, it is easy to identify a set of i40-699 indels that are closely linked to the mutation of interest by spotting a set of neighboring PCR products without smaller CB4856 products. Finally, we used crude lysates using as little as 0.001 adult hermaphrodites for each PCR sample to demonstrate the ease of mapping using these primers.

First, to validate the primers and i40-699 indels for mapping, we mapped three known mutations *clr-1(e1754)*^42^, *sqv-3(n2842)*^46^, and *sqv-5(n3039)*^47^. These mutations were chosen in part because the corresponding homozygous mutants are sterile, which makes it difficult to obtain large amounts of genomic DNA from homozygous mutants. As is standard, all mutants were originally isolated from mutagenized N2 and subsequently were outcrossed with non-mutagenized N2 by others. From heterozygous F1 progeny derived from mating of CB4856 males with mutant hermaphrodites of interest, we obtained individual F2 adult hermaphrodites with Clr or Sqv mutant phenotype. Ten F2 mutants were lysed and pooled for mapping each mutation using 96 primer pairs. Here, we mapped all three mutations to within 6 to 8 Mb intervals by identifying neighboring sets of PCR samples without smaller CB4856 products (Supplementary Figure S2). We did not pursue use of lysates with 0.001 adult hermaphrodites in these examples unlike in the next set of mapping of previously unknown mutations.

We mapped a previously unknown *iw108* mutation present in an MMP strain VC20379. VC20379 is a part of 2,007 whole-genome sequenced strains that had been inbred following mutagenesis^36^, and we found an unknown mutation that is associated with a constitutive temperature-dependent dauer mutant phenotype at 28 °C^45^. Using pooled and individual F2 mutants, we mapped the mutation to the left side of chromosome X using the i40-699 indel detection primer set (Supplementary Figure S3). Within the mapped interval according to MMP sequencing data, there are two point mutations in introns of *tmc-1* and *fbxa-40* along with five point mutations in intergenic regions without any interesting nearby genes. Subsequently, we obtained an outcrossed constitutive dauer mutant isolate with the *tmc-1* mutation but without five of the seven mutations including the *fbxa-40* mutation. We examined *tmc-1(ok1859)* 2025 bp deletion and *tmc-1(gk631913)* Q578amber mutants, but these two mutants were without dauer mutant phenotype. After exhausting other possibilities including genome-wide examination of all mutations with moderate or strong effect on protein coding in VC20379 as reported by MMP, we performed de novo analysis of the raw VC20379 MMP sequencing data using a standard pipeline of bowtie2^48^, samtools^49^, vcftools^50^, and snpEff^51^. To the left of the aforementioned seven mutations, we identified an unreported mutation *iw108*, which is predicted to cause a G-to-T missense mutation causing C848F change in the transcript F25E2.5a.1 of *daf-3* SMAD4. The aforementioned outcrossed mutant isolate with the *tmc-1* mutation but not *fbxa-40* mutation contains *daf-3(iw108)*. Curiously, *daf-3(iw108)* mutation is identical to *daf-3(gk269916)* reported to be present in another MMP strain VC20208. The *daf-3(gk269916)* mutation is absent in VC20208, and our best guess is that this is a WormBase annotation error. Mutations in *daf-3*^52^ are associated with dauer defective phenotype at standard growth temperatures^53^ and with dauer constitutive phenotype at temperatures above 25 °C^54^. We also have examined the canonical *daf-3(e1376)* mutant and a previously uncharacterized *daf-3(ok3610)* mutant, both of which have a similar temperature-dependent dauer mutant phenotype as *daf-3(iw108)* and support the notion that *daf-3* is the gene of interest. Finally, we found that *daf-3(ok3610)* and *daf-3(iw108)* mutations complement each other to show a temperature-dependent dauer constitutive mutant phenotype. Investigation of *daf-3(iw108)* is an example of mapping of unknown mutation, mutation identification, and confirmation with additional mutant alleles with the same mutant phenotype and complementation test.

Next in a *daf-3(iw108)* mutant strain, we identified a previously unknown *iw109* mutation associated with a temperature-dependent constitutive dauer mutant phenotype at 28 °C. We found the *iw109* mutation while trying to obtain nice images of *daf-3* pooled mapping, which did not result in the expected mapping of *daf-3(iw108)* to the left side of chromosome X. Instead, we mapped *iw109* to chromosome II using an outcrossed *iw109* strain without *daf-3(iw108)* (Supplementary Figure S4). The *iw109* mutation is associated with a stronger dauer constitutive mutant phenotype as compared to *daf-3(iw108)*, whose molecular lesion had been known to us for two years by the time of our discovery of *iw109*. We presume that *iw109* mutation arose spontaneously and became fixed during two years of culturing of *daf-3(iw108)* mutants. Whole-genome sequencing of *iw109* mutants revealed a 29 bp deletion mutation with the loss of DNA sequence TTTTTGCAAAGTTGAAAGAGGAATTGTTC in the second exon of *che-10* ciliary rootlet coiled-coil (CROCC) or rootletin^55^ in the mapped region. Here we expect only 168 amino acids of four isoforms of full-length CHE-10 proteins ranging from 1575 to 1995 amino acids to be retained. The sequencing also revealed a ~ 1.3 kb deletion removing a part of nearby *hmg-11* gene and upstream DNA, but we think that this deletion is unlikely to be responsible for the dauer mutant phenotype. Notably, *che-10(e1809)* mutants are defective in differentiation of amphid neurons and dauer formation at normal temperature under 25 °C^56^, and these mutant phenotypes are associated with dauer constitutive mutant phenotype only at above 25 °C^54,57^. Furthermore, we used the *che-10(e1809)* mutation for a complementation test with *iw109*, and these two mutations complement each other to show a dauer constitutive mutant phenotype at 28 °C. Our studies starting with mapping suggest that *iw109* could be a *che-10* null mutation.

Third, we identified previously unknown *iw107* mutation associated with a temperature-dependent constitutive dauer mutant phenotype at 28 °C. We found *iw107* mutation in PR691 *tax-2(p691)*, which is called RS1 in older manuscripts^58–60^, during outcrossing. Specifically, we obtained *iw107* mutants without *tax-2(p691)* mutation as well as *tax-2(p691)* mutants by screening for temperature-dependent dauer mutant descendants following mating of PR691 with N2 wild type. We think that *iw107* mutation may have arisen spontaneously and become fixed in PR691, possibly prior to our receipt of PR691 from the Caenorhabditis Genetics Center. We mapped *iw107* to the left side of chromosome X using pooled and individual F2 mutants (Supplementary Figure S5). Within the mapped interval, there are five genes *che-2*^61^, *daf-3*^52^, *dyf-11*^62,63^, *osm-5*^64^, and *rund-1*^65^, which by our assessment could have a temperature-dependent constitutive dauer mutant phenotype. We performed complementation tests using mutants with lesions in these genes, and among these only *che-2(e1033)* Q601ochre^61^ and *iw107* complemented each other to show a lasting dauer constitutive mutant phenotype at 28 °C. Sanger sequencing of *iw107* mutant did not reveal any mutation in the coding region of *che-2*, but we identified mutations in two different intervening introns. Specifically in the *che-2* intraflagellar transport 80 (IFT80^66^) transcript F38G1.1.1 unspliced and untranslated (UTR) sequence of 5055 bp, the two mutations are G-to-A at positions 2750 and 3864 with the latter mutation near the splice donor of exon 11 (GA/gtgagt to GA/gtgaat with slash denoting exon/intron boundary and underline denoting the site of sequence change). In addition to *che-2(iw107)* mutants having fewer dauer survival at 28 °C as compared to *che-2(e1033)* mutants, we saw that *che-2(iw107)* mutants do not show strong male mating defect observed with *che-2(e1033)* mutants^67,68^, which is consistent with *iw107* being a weak loss-of-function mutation. In summary, *iw107* appears to be a weak *che-2* loss-of-function mutation as shown by mapping and other studies.

### Easily distinguished indels in other wild isolates

While neither CB4856 nor N2 wild-type strains shows constitutive dauer mutant phenotype, hybrid progeny resulting from mating between CB4856 and N2 could survive as dauer larvae for a long time at high temperature^45^. The dauer survival phenotype of the hybrid is very weak and usually is not reproducible using the progeny of the survivors, and thus CB4856 still could be used for mapping with sufficient caution. In general, however, a common phenotype shared between the mutant of interest and CB4856 could render CB4856 unsuitable for use in mapping. For example, CB4856 displays social feeding behavior similar to mutants of neuropeptide receptor *npr-1*^69^ and thus would be inappropriate for mapping mutations associated with such feeding behavior. Here, another wild isolate without the same social feeding behavior is needed. To assess other wild isolates for use with i40-699 indel mapping, we examined the 40 wild isolates sequenced by the MMP^36^ with the vast majority of reported i40-699 indels. While there is whole-genome sequence data of over 300 wild isolates available elsewhere in the *C. elegans* natural diversity resource CeNDR^70^, bigger indels were identified at low rate by CeNDR. For example with CB4856, only two indels of > 40 bp were identified by CeNDR using higher threshold for quality control as opposed to over 1,600 i40-699 indels according to MMP. Thus to identify more useful wild isolates for mapping, we only examined the distribution of over 11,000 i40-699 indels in the 40 MMP wild isolates.

**Figure 4:**
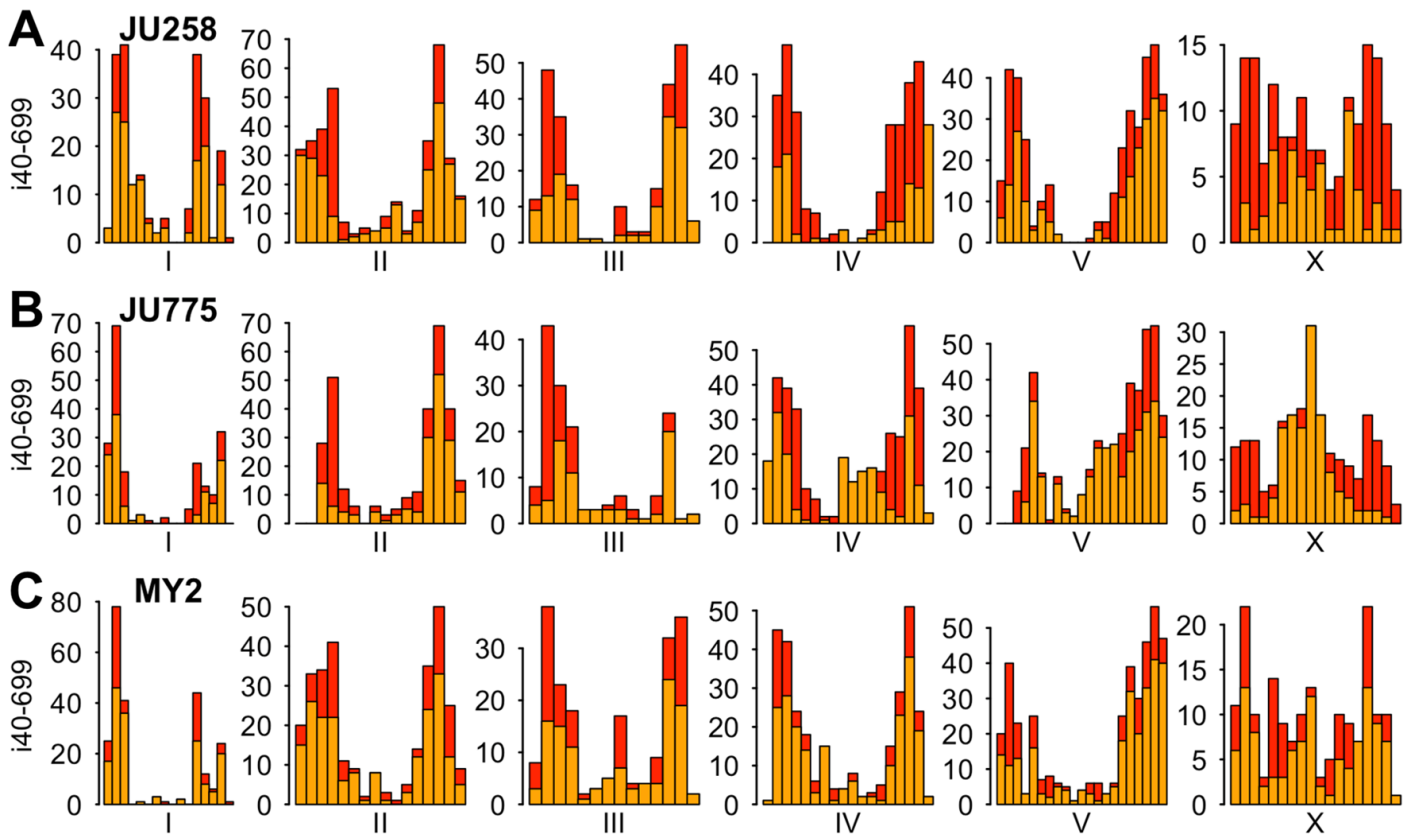
Three more wild isolates with many i40-699 indels. Shown are distributions of i40-699 indels in 1 Mb intervals of wild isolates (A) JU258, (B) JU775, and (C) MY2. Orange bars indicate i40-699 indels that are unique among the four wild isolates JU258, JU775, MY2, and CB4856. Red bars indicate i40-699 indels that are also present in other wild isolates.

A suitable alternate wild isolate should be evolutionarily divergent from CB4856 and therefore is less likely to share a common phenotype with CB4856. Also, a good alternate should contain many i40-699 indels throughout the genome. We discarded 34 of the 40 wild isolates from consideration because their genomes have too many 1 Mb intervals without any i40-699 indels (Supplementary Figure S6). We note that these 34 wild isolates contain over 6000 unique i40-699 indels combined, and some of these wild isolates could be used for mapping small parts of the genome. Of the others, three isolates JU1171, MY2, and MY14 share most of their i40-699 indels evenly throughout the genome (Supplementary Figure S7), meaning they are closely related to each other as also noted previously^39,71^. Such closely related wild isolates are likely to share common phenotypes, and thus we chose MY2 to represent this group of three. This leaves us with the three best wild isolates JU258, JU775, and MY2 all with i40-699 indels in most but not all 1 Mb intervals (Fig. 4). We recommend using JU258, JU775, and MY2 for building a similar mapping toolkit using a different wild isolate, and we provide a list of over 11,000 potentially useful i40-699 indels with annotations in Supplementary Table S1.

## Discussion

Using i40-699 indels, mapping could be done quickly and easily. Pooled mapping could be completed on the day of F2 mutant collection, and additional fine mapping could be completed the next day if needed. The amount of time required on temperature cyclers and electrophoresis apparatus include 1 h 20 min for single worm lysis, 2 h 20 min for PCR, and 40 min for agarose gel electrophoresis. Time needed for transfer of 10 N2 and 10 mutant adults for lysis, preparation of two sets of 96 PCR reactions for whole-genome mapping, and gel loading of PCR products could be under one hour with experienced hands and good equipment. With other mapping methods, five or more days are needed to collect large populations of worms derived from F1 mutant worms before starting subsequent procedures. No need to grow many worms is a big advantage with the primers we designed for the i40-699 indel mapping. Thus mapping using i40-699 indels is faster than all existing mapping methods.

Mapping is a part of many experiments necessary to link a mutation in a gene to a mutant phenotype. Other experiments could involve complementation test, rescue, or identification of many mutant alleles in the same gene with the same mutant phenotype. A key requirement is the identification of the molecular lesion in the DNA, which requires DNA sequencing. Of existing mapping methods, combining whole-genome-sequencing and mapping is especially attractive, and a comparison with i40-699 indel mapping could be worthwhile. With speed, i40-699 indel mapping is faster. With resolution, mapping with whole-genome-sequencing is usually better. With cost, we discuss the parameters only because the exact cost is neither static nor global. Here, a consideration is that whole-genome-sequencing mapping could involve different modern approaches^72,73^, which may or may not involve pooling. With pooling, 20 to 50 populations derived from F2 mutants need to be grown before pooling for DNA purification, library construction, and sequencing run in addition to extra reagents for culturing worms. Without pooling, there is a need for separate DNA purifications, library constructions, and more sequencing runs or space. With i40-699 indel mapping, there are extra supplies and reagents, such as PCR plates, pipette tips, Taq polymerase, dNTPs, and primers. Assuming that whole-genome sequencing is needed, combining whole-genome sequencing and mapping using pooling probably would be the cheapest option with a single mutation. We note that with i40-699 indel mapping and with many mutations, prior complementation tests could reduce the number of needed whole-genome sequencing runs, and the use of Sanger sequencing to identify mutations could lower the cost. In our opinion, i40-699 indel mapping is a valid fast option.

**Figure 5:**
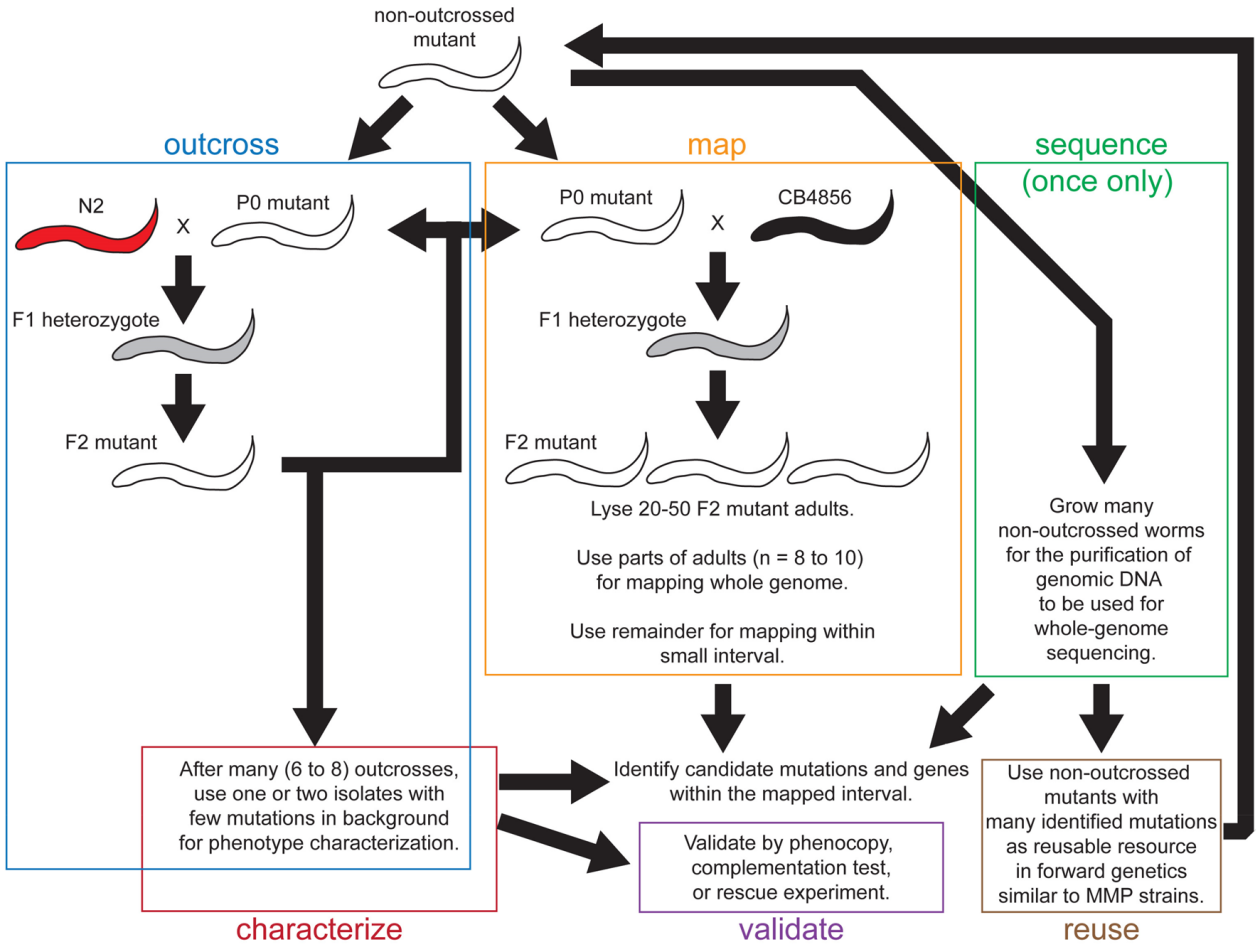
Using i40-699 indel mapping with outcrossing and sequencing for gene identification, mutant characterization, and reusable forward genetics resource. Outcrossing could eliminate most background mutations, which could interfere with the mutant phenotype. Mapping and sequencing also could help pinpoint a mutation to a gene. Validations could be provided by complementation test, rescue experiments, or many mutations in the same gene, which phenocopy the mutation of interest. We recommend performing whole-genome sequencing using non-outcrossed mutant strain, which could be re-used for more forward genetics. No new whole-genome sequencing is needed with MMP strains. White worm is mutant, black worm is CB4856 HA, red worm is N2 wild type, and gray worm is heterozygous.

We also discuss a special circumstance, which should be more common in our opinion. Pre-existing whole-genome-sequencing data exists with the 2007 MMP strains^36^, which we think are a very useful resource for the *C. elegans* scientific community. In this manuscript with i40-699 indel mapping, we did not need a new whole-genome sequencing to identify *daf-3(iw108)* with MMP strain VC20208. Similarly, 2007 MMP strains could be used for future genetic screens and identification of interesting mutations without new whole-genome sequencing. Notably following mutagenesis and genetic screen, new mutants with whole-genome sequencing data could have the same utility as the MMP strains. Here prior to sequencing, outcrosses removing unlinked mutations is counterproductive, but inbreeding could be useful. With i40-699 indel mapping, whole-genome-sequenced mutants could be added to the useful existing scientific resource consisting of the 2007 MMP strains. On the other hand with the use of whole-genome sequencing for mapping, the sequenced worms are not useful because they have either too few mutations or a confounding mix of CB4856 and N2 DNA. In summary, the use of i40-699 mapping could be used as a part of a general effort to improve the *C. elegans* community resource for forward genetics (Fig. 5).

For efficient use of i40-699 indel primers with mutant lysates with a mix of N2 and CB4856 DNA, we recommend using 0.01 adults for each PCR reaction. While it is possible to use 0.001 adults per PCR sample (Supplementary Figures S3 to S5), PCR success rate is lower at such low template concentration, and there could be more off-target PCR products. Strong off-target PCR products could make the results more difficult to interpret. We also note that the use of a smaller subset of primer pairs (e.g. 48 instead of 96) for whole-genome mapping could be more cost-effective. Here, we suggest picking primer pairs based on high PCR success rate using less CB4856 DNA (Supplementary Table S2). Some of the alternative primer pairs described in Supplementary Table S2 other than the most extensively tested 102 pairs may prove to be more reliable. Also, we recommend using a small number (e.g. 10) of F2 mutant adults for pooling to avoid unnecessary dilution. With the mutation of interest mapped to a small interval, primer pairs for detecting a subset of 102 i40-699 indel loci could be used for further mapping using individual lysates of F2 mutants (Fig. 5).

To assess how much mapping may be needed, we examined MMP whole-genome sequencing data of 2,007 mutagenized strains^36^ for the genomic distribution of mutations. MMP used six mutagenesis methods, and MMP obtained the largest number of mutations with 50 mM EMS, which is commonly used in many laboratories, and with the combination of 50 mM EMS and 1 mM ENU (Supplementary Table S4). With 50 mM EMS alone (n = 656), MMP identified 376 ± 100 (mean ± s.d.) mutations, of which 86 ± 24 mutations are predicted to have an effect. Here, the following search terms were used as a prediction of an effect: nonsense, start ATG, missense, frame shift, coding exon, across exon boundary, splicing, miRNA, rRNA, scRNA, snRNA, snoRNA, and tRNA. While there are many mutations far away from exons known to have substantial biological effects, we expect that most mutations of intergenic region, intron, non-coding exon, UTR, and ncRNA have no effect. With the combination of 50 mM EMS and 1 mM ENU (n = 943), MMP identified 545 ± 149 mutations, of which 123 ± 34 mutations are predicted to have an effect. Laboratory-induced mutations are evenly distributed throughout the genome. Given the *C. elegans* genome of just over 100 Mb, a typical mutagenized strain using EMS alone has on average less than one mutation with a predicted effect per Mb. With primer pairs for i40-699 indel in most 1 Mb intervals, narrowing the number of strong candidates to one should be possible in many cases with i40-699 indels.

Mapping using i40-699 indels and small number of worms is fast and easy for a typical laboratory and does not need expensive specialty equipment. In planning a comprehensive strategy for research, optimal use of all necessary methods is desired. Among key techniques used in genetics, recently developed methods include whole-genome sequencing and CRISPR/Cas9. Combining i40-699 indel mapping with whole-genome sequencing and other methods as described here could be a part of comprehensive strategy for forward genetics (Fig. 5).

## Supplementary information

Supplementary Information 1.

Supplementary Information 2.

Supplementary Information 3.

Supplementary Information 4.

Supplementary Information 5.

## Data Availability

Additional material including strains and scripts are available upon request.
