# Supplementary material for: Fast genetic mapping using insertion-deletion polymorphisms in Caenorhabditis elegans
Source: Sci Rep. 2021 May 26;11:11017. doi: 10.1038/s41598-021-90190-x (PMC8155061; doi:10.1038/s41598-021-90190-x)
Supplement: Supplementary file 1 — Supplementary Information 1. [file 41598_2021_90190_MOESM1_ESM.pdf]

Fast genetic mapping using insertion-deletion polymorphisms in *Caenorhabditis elegans*

Ho-Yon Hwang and Jiou Wang

Supplementary Material

Supplementary Table S1. All i40-699 indels in the 40 wild isolates. Shown in order by column are description of the i40-699 indels including WormBase ID, mutation type, chromosome, start and end of mutation, bp in N2 reference but not in another wild isolate, bp not in N2 reference but in another wild isolate, bp change from N2 reference, nearby gene, genetic position of the gene, left and right ends of the gene, frequency of appearance of the i40-699 indel among 40 wild isolates, list of strains with the i40-699, and other descriptions.

Supplementary Table S2. Primer pairs and i40-699 indels with extensive testing. Shown in order by column are chromosome and start and end of i40-699 indel, position within the 96-well plate used for mapping in this manuscript, WormBase ID, MMP ID, size of deletion and insertion and overall change, primer sequences, sizes of PCR products corresponding to N2 and CB4856, PCR success rate in percentages using 0.1 adults per PCR with the number of trials (n) in parenthesis, PCR success rate in percentages for CB4856 products using 0.01 CB4856 adults and 0.09 N2 adults with the number of trials (n) in parenthesis, nearby gene with their genetic and physical position, frequency of appearance among 40 wild isolates, and list of wild isolates with the i40-699 indel.

Supplementary Table S3. Primers used to examine i40-699 indels. Shown in order by column are MMP ID, WormBase ID, chromosome and start of i40-699 indel, primer ID, primer sequences, and description of primer quality. For description of primer quality, the best primers are one of the primers

for 102 i40-699 indel loci listed in Supplementary Table S2, and the other primers are described using terms ok, weak, and poor in decreasing order of quality.

Supplementary Table S4. Summary of mutations in 2007 MMP mutagenized strains by mutagenesis methods. Shown in order by column are the mean number of mutations and standard deviation, median and maximum number of mutations, the mean number of mutations with stronger possibility of effect and standard deviation, median and maximum number of such mutations with possible effect, and the number of mutagenized strains obtained. These summaries are provided for six different mutagenesis methods used.

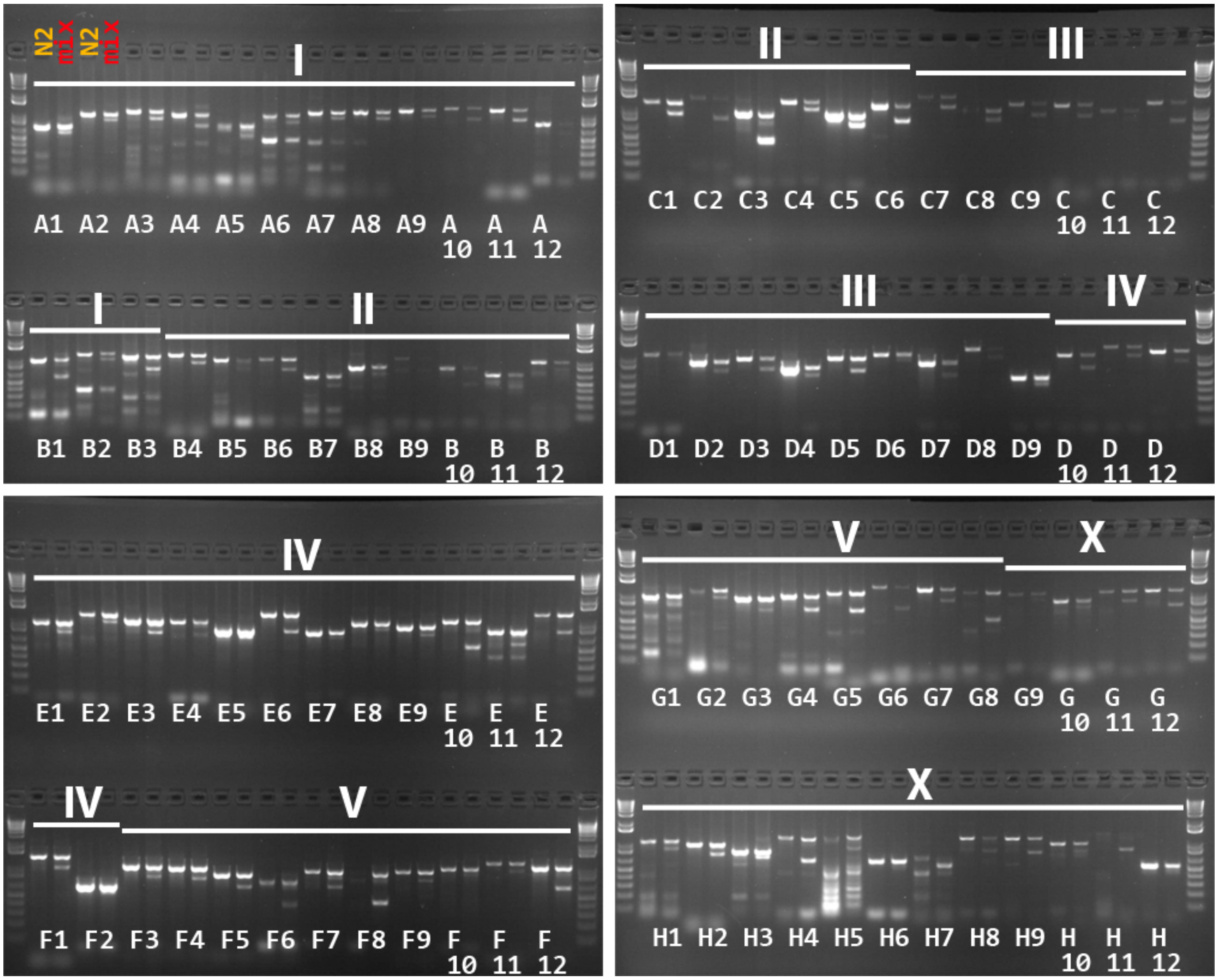

Supplementary Figure S1. Using the primer set with smaller amount of CB4856 DNA. Pairs of PCR products using DNA from N2 wild type on left and from nine parts N2 and one part CB4856 on right. The samples are arranged in 8 rows from A to H with each row containing 12 different primer pairs. Shorthand designations from A1 to A12 through from H1 to H12 indicate primer pair positions in a 96-well plate. Chromosomes are indicated as I, II, III, IV, V, and X. 1 kb Plus Ladder Invitrogen.

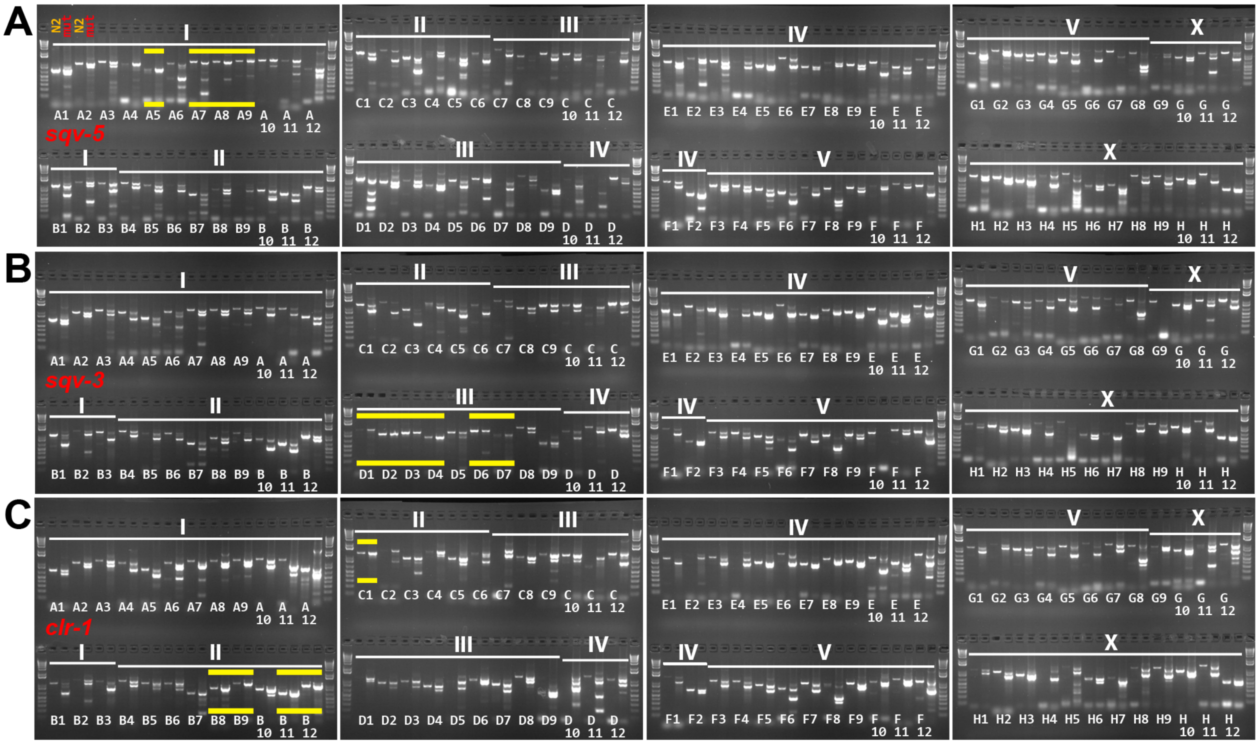

Supplementary Figure S2. Using the i40-699 indel detection primers for mapping mutations already associated with genes. Pairs of PCR products using DNA from N2 wild type on left and from mix of mutant F2 on right. The mutants are (A) *sqv-3(n2842)*, (B) *sqv-5(n3039)*, and (C) *clr-1(e1754)*. For each mapping, ten F2 mutant adults and ten N2 adults were lysed individually in 10 µl. While keeping the mutant and N2 samples separate, the ten lysates were combined and were diluted with 35 µl of water, and 1 µl of the diluted mix was used for each PCR sample. Each PCR used lysates with less than 0.1 adults. For each mutation, closely linked i40-699 indels without CB4856 PCR products are highlighted with yellow bars. The samples are arranged in 8 rows from A to H with each row containing 12 different primer pairs. Shorthand designations from A1 to A12 through from H1 to H12 indicate primer pair positions. Chromosomes are indicated as I, II, III, IV, V, and X. 1 kb Plus Ladder Invitrogen.

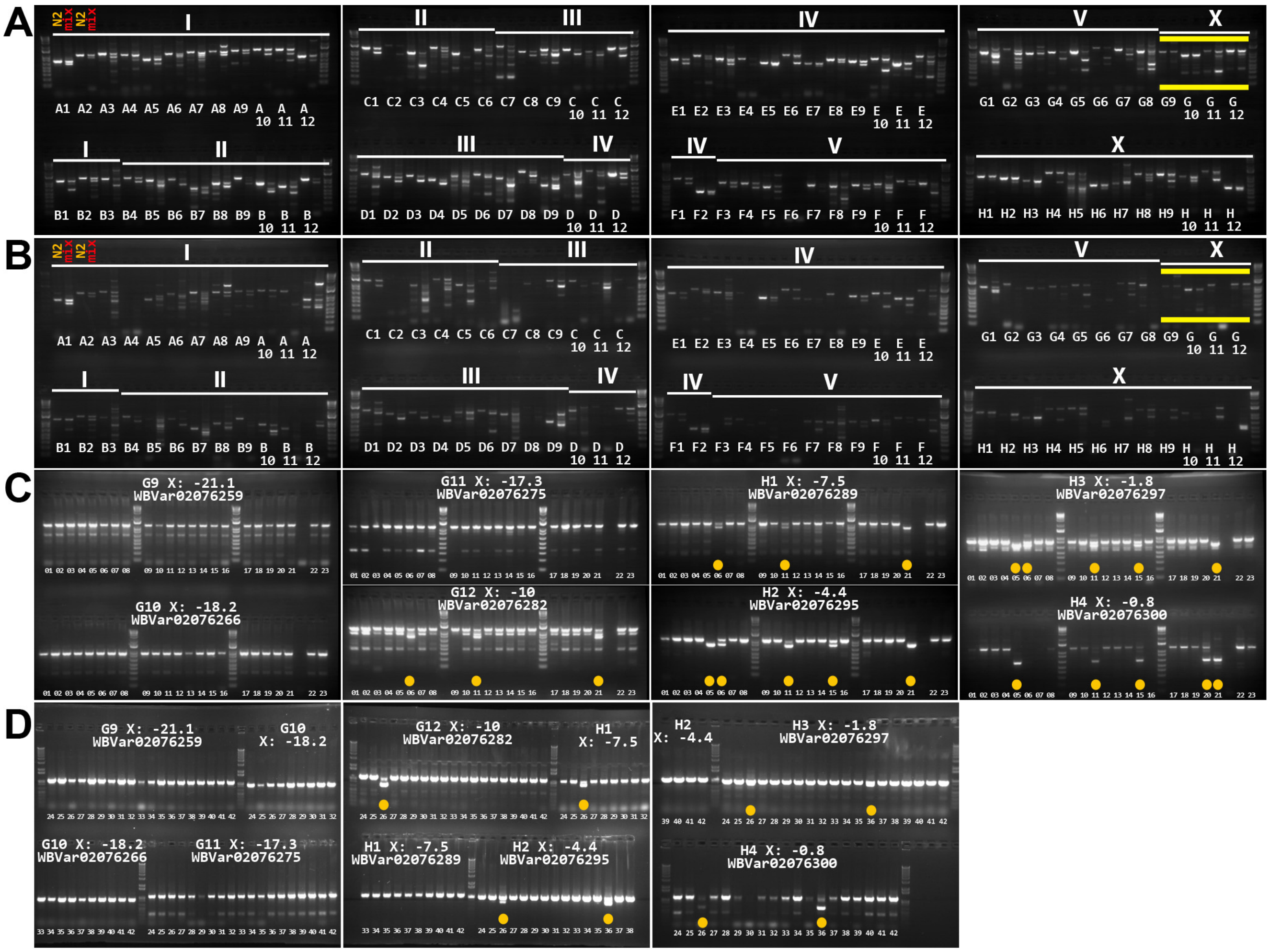

Supplementary Figure S3. Using the i40-699 indel detection primers for mapping previously unknown mutation *daf-3(iw108)*. (A) Pairs of PCR products using DNA from N2 wild type on left and from mix of *daf-3(iw108)* mutant F2 on right. Either ten N2 adults or ten mutant adults were lysed individually in 10 µl, and 1 µl from each lysate was used in preparation of master mix for 100 PCR samples. Thus, each PCR used DNA from approximately 0.01 adults as template. Closely linked i40-699 indels without CB4856 PCR products are highlighted with yellow bars (G9 to G12). The samples are arranged in 8 rows from A to H with each row containing 12 different primer pairs. Shorthand designations from A1 to A12 through from H1 to H12 indicate primer pair positions. See Supplementary Table 2 for additional information on primer pairs for H4 and H7. Chromosomes are indicated as I, II, III, IV, V, and X. 1 kb Plus Ladder Invitrogen. (B) We used the lysates from the same adults as (A) except 0.1 µl from each lysate was used to prepare the master mix. Thus, each PCR used approximately 0.001 adults. See Supplementary Table 2 for additional information on primer

pairs for H4, H5, and H7. (C) Results of PCR using individual mutant lysates 01 to 23. Each mutant was individually lysed in 10 μl. Either 0.1 μl of undiluted lysate or 1 μl of 10-fold diluted lysate with approximately 0.01 adults for each PCR. Orange circles indicate F2 mutants with smaller PCR products corresponding to CB4856. (D) Results of PCR using individual mutant lysates 24 to 42. For each lysate, 5 F1 descendant adults derived from parental worms that survived as dauer larvae for 10 days at 28°C were lysed in 10 μl, and 1 μl of the lysate with approximately 0.5 adults was used for each PCR. Here, 1% agarose gel was used instead of 2% agarose in (A-C).

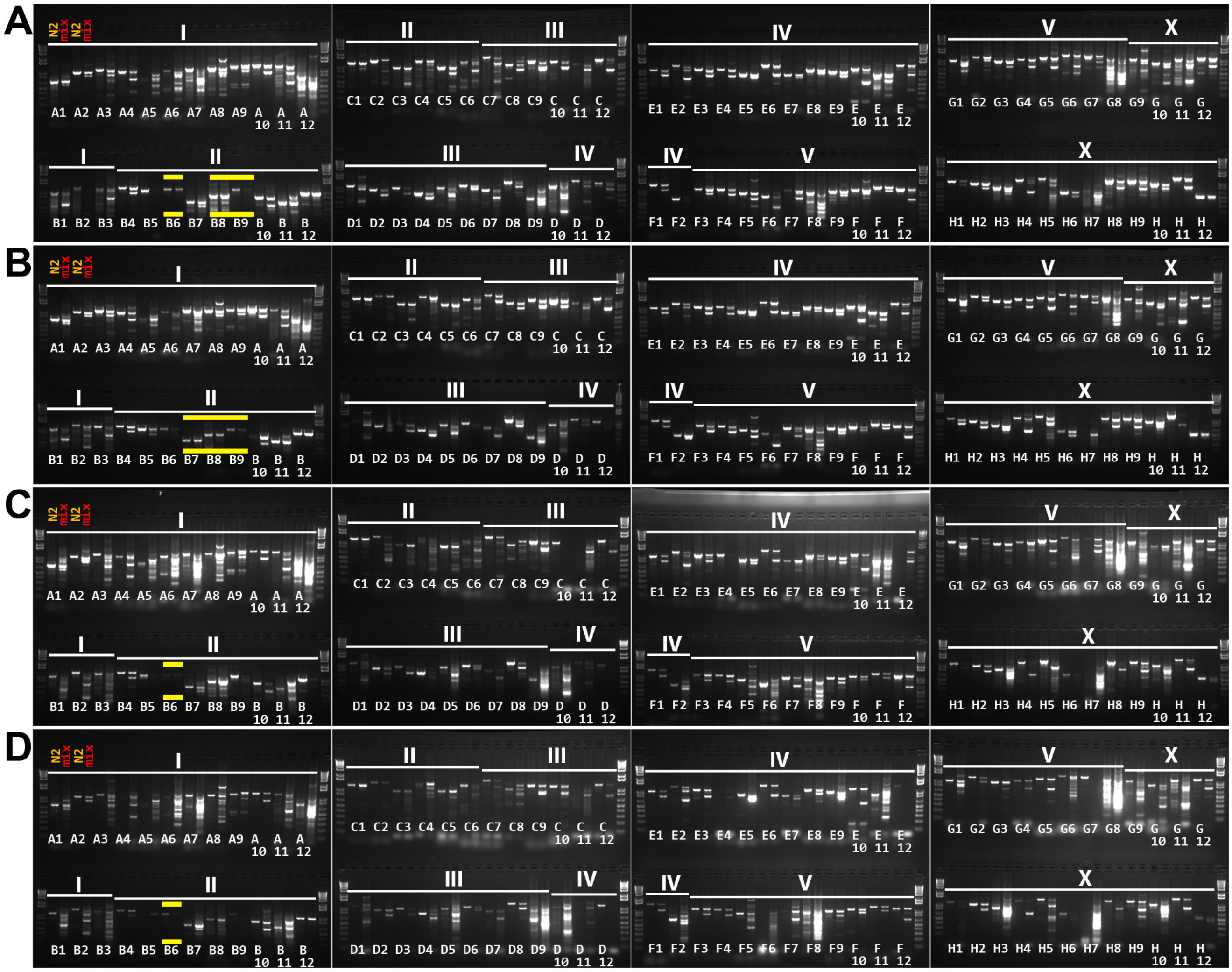

Supplementary Figure S4. Using the i40-699 indel detection primers for mapping previously unknown mutation *che-10(iw109)*. (A) Pairs of PCR products using DNA from N2 wild type on left and from mix of mutant F2 on right. Here, PCR master mix was prepared without primers and template. Dilution of combined lysate samples with DNA template was made using 1x lysis buffer without proteinase K. For N2, four adults were lysed individually in 10 μl each and were combined and diluted with 70 μl of lysis buffer. For *iw109*, 14 adults were lysed individually, and 3 μl aliquots from each were combined and diluted with 70 μl of lysis buffer. One μl was used per PCR, meaning each PCR used approximately 0.04 adults. Closely linked i40-699 indels without CB4856 PCR products are highlighted with yellow bars. The samples are arranged in 8 rows from A to H with each row containing 12 different primer pairs. Shorthand designations from A1 to A12 through from H1 to H12

indicate primer pair positions. Chromosomes are indicated as I, II, III, IV, V, and X. 1 kb Plus Ladder Invitrogen. (B) Same as (A) with following changes. For N2, two adults were lysed individually and were combined and diluted with 120 µl of lysis buffer. For *iw109*, 16 adults were lysed individually, and 1 µl from each were combined and diluted with 95 µl of lysis buffer. One µl was used per PCR, meaning each PCR used approximately 0.01 adults. (C) Same as (A) with following changes. For N2, four adults were lysed individually, and one µl from each were combined and diluted with 95 µl of lysis buffer. For *iw109*, 16 adults were lysed individually, and 0.2 µl from each were combined and diluted with 95 µl of lysis buffer. One µl was used per PCR, meaning each PCR used approximately 0.003 adults. (D) Same as (A) with following changes. For N2, four adults were lysed individually, and 0.3 µl from each were combined and diluted with 120 µl of lysis buffer. For *iw109*, 12 adults were lysed individually, and 0.1 µl from each were combined and diluted with 120 µl of lysis buffer. One µl was used per PCR, meaning each PCR used approximately 0.001 adults.

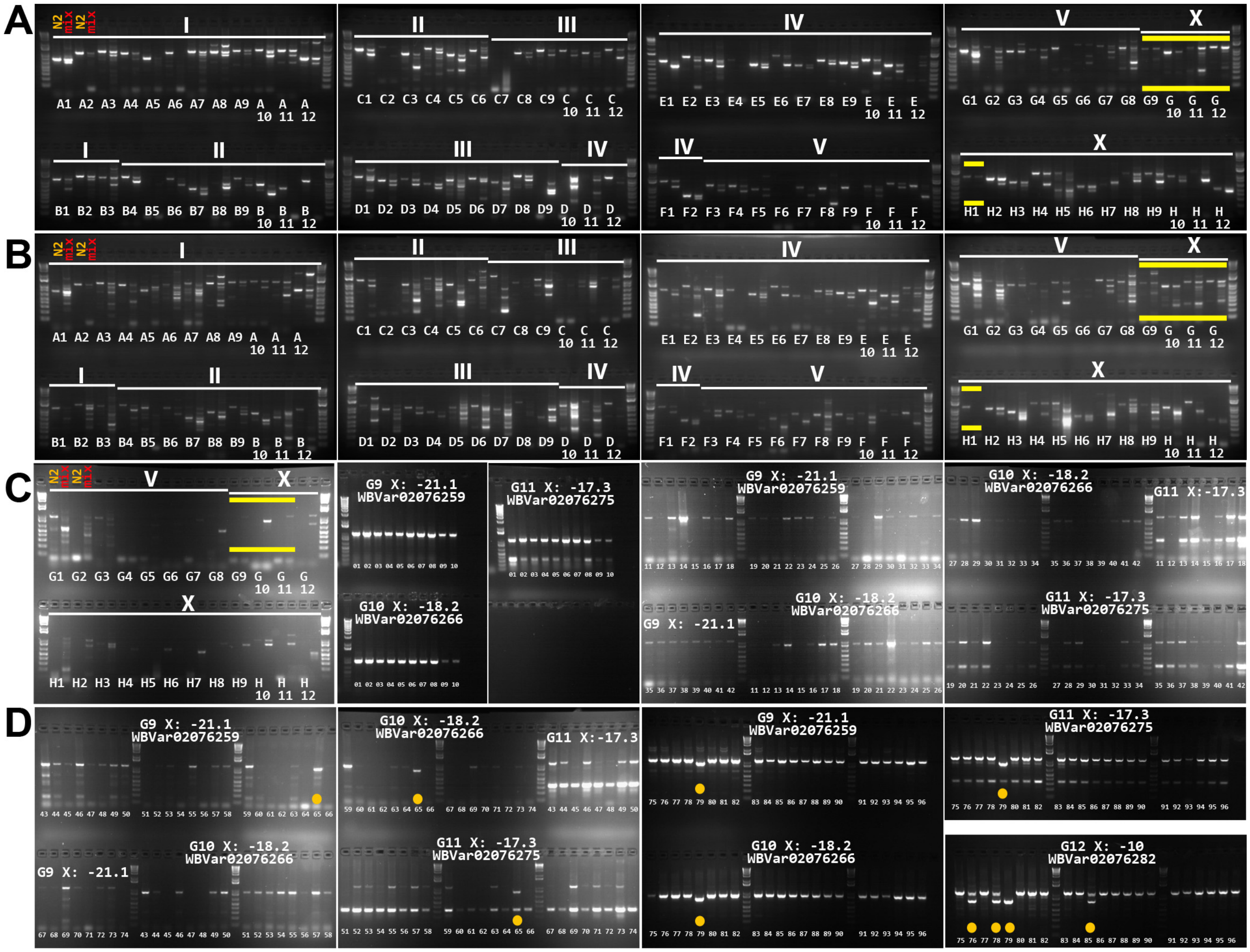

Supplementary Figure S5. Using the i40-699 indel detection primers for mapping previously unknown mutation *che-2(iw107)*. (A) Pairs of PCR products using DNA from N2 wild type on left and from mix of *che-2(iw107)* mutant F2 on right. Either ten N2 adults or ten mutant adults were lysed individually in 10 µl, and 1 µl from each lysate was used in preparation of a master mix for 100 PCR samples. Thus, each PCR used DNA from approximately 0.01 adults as template. Closely linked i40-699 indels without CB4856 PCR products are highlighted with yellow bars. The samples are arranged in 8 rows from A to H with each row containing 12 different primer pairs. Shorthand designations from A1 to A12 through from H1 to H12 indicate primer pair positions. See Supplementary Table 2 for additional information on primer pairs for H4 and H7. Chromosomes are indicated as I, II, III, IV, V, and X. 1 kb Plus Ladder Invitrogen. (B) We used the lysates from the same adults as (A) except 0.1 µl from each lysate was used to prepare the master mix. Thus, each PCR used approximately 0.001

adults. See Supplementary Table 2 for additional information on primer pairs for H4 and H7. (C) Leftmost panel shows a part of a mapping effort using i40-699 indel detection primers. Here, PCR master mix was prepared without primers and template. Either ten N2 adults or ten *iw107* mutant adults were lysed individually in 10 µl and were combined and diluted with 100 µl of 1x lysis buffer without proteinase K. We used 1 µl of diluted lysate per PCR, meaning each PCR used approximately 0.05 adults. This data suggests that i40-699 indel corresponding to position G12 is not closely linked to *che-2(iw107)*. For next two panels in the second quartile with samples labeled 01 to 10, lysates were prepared using F1 descendant adults of the ten *iw107* mutant adults used in the first panel. Here, eight F1 adults were used for each lysate preparation in 10 µl, and 1 µl of the lysate with approximately 0.8 adults was used for each PCR. The two panels on right show results of PCR using single mutant lysates 11 to 42. Each mutant was individually lysed in 10 µl, and 1 µl of the lysate with approximately 0.1 adults was used for each PCR. We think that poor PCR in (C) was caused by an old partially degraded 1x lysis buffer. Lysate preparations were made over the course of more than a month. Specifically, lysis for the samples 01 to 10 in two small panels on the second quartile was done 2-3 days after the lysis for the samples used in the leftmost first panel, and lysis for the samples 11 to 42 in the right two panels was done 19-20 days later. Orange circles indicate F2 mutants with smaller PCR products corresponding to CB4856. (D) Results of PCR using single mutant lysates 43 to 96. Each mutant was individually lysed in 10 µl, and 1 µl of the lysate with approximately 0.1 adults was used for each PCR. Lysis for the samples 43 to 74 in the left two panels was done one day after the lysis for the the samples 11 to 42 in (C) using the same lysis buffer, and lysis for the samples 75 to 96 in the right two panels was done after another 25 days using a different newly prepared 1x lysis buffer. Aberrant results of two samples 65 and 79 suggesting no linkage to the left side of chromosome X could be a consequence of an artifact. The suspected artifact is a low-penetrance dauer survival phenotype in hybrid progeny between CB4856 and N2. Because this hybrid phenotype[45] was identified long after the relevant samples were discarded, we were unable to verify that the conflicting results are consequences of the hybrid phenotype.

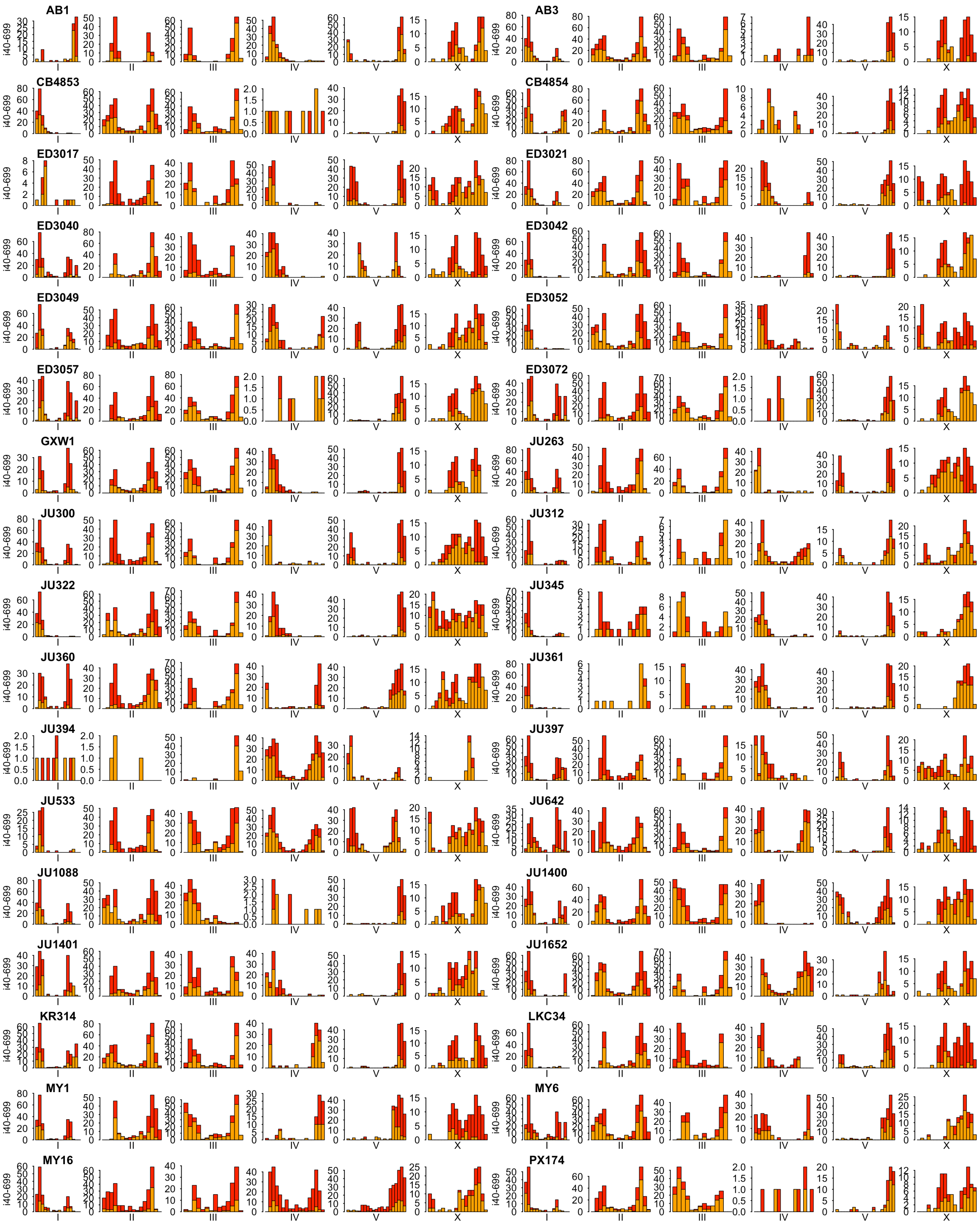

Supplementary Figure S6. Distribution of i40-699 indels in other whole-genome-sequenced wild isolates. Shown are distributions of i40-699 indels in 1 Mb intervals of 34 wild isolates. Red bars indicate i40-699 indels that are also present in the wild isolates CB4856, JU258, JU775, and MY2. Orange bars indicate i40-699 indels that are not present in the wild isolates CB4856, JU258, JU775, and MY2.

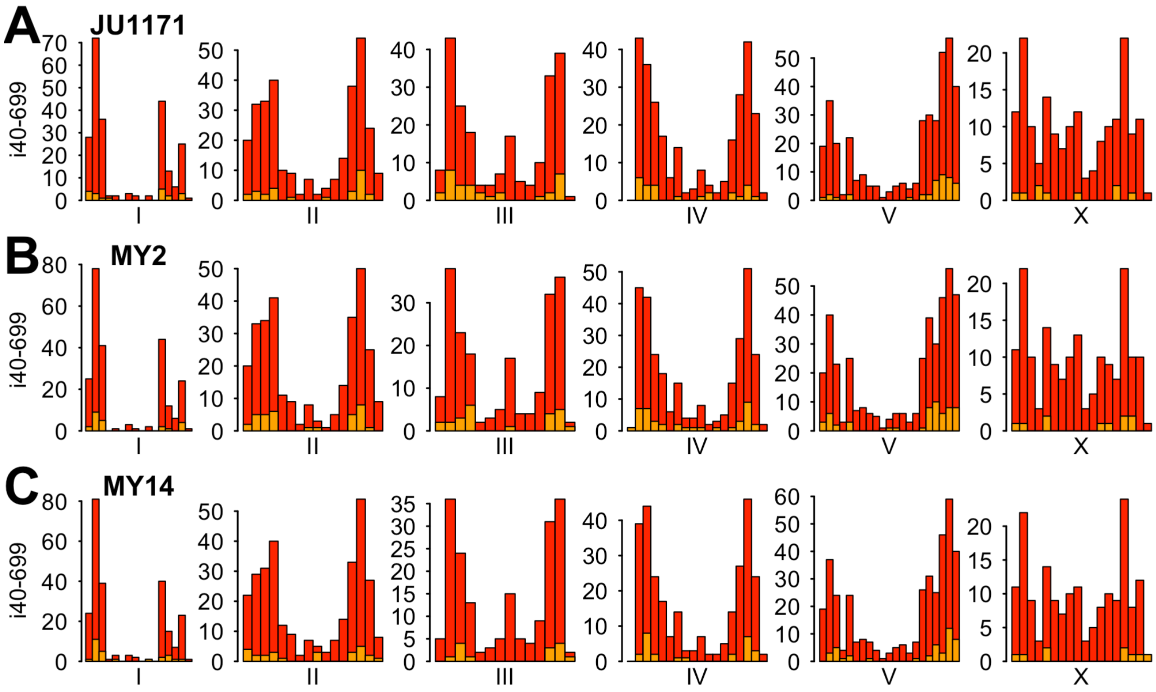

Supplementary Figure S7. Three closely related whole-genome sequenced wild isolates.

Shown are distributions of i40-699 indels in 1 Mb intervals of (A) JU1171, (B) MY2, and (C) MY14. Red bars indicate i40-699 indels that are present in the other two wild isolates. Orange bars indicate i40-699 indels that are unique among the three wild isolates.
